# Peach fruit PpNAC1 activates *PpFAD3-1* transcription to provide *ω*-3 fatty acids for the synthesis of short-chain flavor volatiles

**DOI:** 10.1093/hr/uhac085

**Published:** 2022-04-04

**Authors:** Zhengnan Jin, Jiaojiao Wang, Xiangmei Cao, Chunyan Wei, Jianfei Kuang, Kunsong Chen, Bo Zhang

**Affiliations:** 1 Laboratory of Fruit Quality Biology, Zhejiang Provincial Key Laboratory of Horticultural Plant Integrative Biology, Zhejiang University, Zijingang Campus, Hangzhou 310058, China; 2 School of Agriculture and Biology, Shanghai Jiao Tong University, Minhang Campus, Shanghai 200240, China; 3 Guangdong Key Laboratory for Postharvest Science, College of Horticultural Science, South China Agricultural University, Guangzhou 510642, China

## Abstract

Volatile organic compounds (VOCs) derived from fatty acids are major contributors to fruit flavor and affect human preferences. The *ω*-3 fatty acid linolenic acid 3 (18:3) serves as an important precursor for synthesis of (*E*)-2-hexenal and (*Z*)-3-hexenol. These short-chain C6 VOCs provide unique fresh notes in multiple fruit species. Metabolic engineering to improve fruit aroma requires knowledge of the regulation of fatty acid-derived VOCs. Here, we determined that ripe fruit-specific expression of *PpFAD3-1* contributes to 18:3 synthesis in peach fruit. However, no significant increases in (*E*)-2-hexenal and (*Z*)-3-hexenol were detected after overexpressing *PpFAD3-1*. Interestingly, overexpressing the *PpNAC1* transcription factor increased the content of 18:3 and enhanced the production of its derived volatiles. Moreover, induced expression of genes responsible for downstream VOC synthesis was observed for transgenic tomato fruit overexpressing *PpNAC1*, but not for transgenic fruit overexpressing *PpFAD3-1*. Electrophoretic mobility shift and ChIP-Seq assays showed that PpNAC1 activated *PpFAD3-1* expression via binding to its promoter. Therefore, *PpNAC1* plays an important role in modulating fatty acid flux to produce fruit flavor-related VOCs. In addition to PpNAC1, *PpFAD3-1* expression was also associated with epigenetic modifications during peach fruit ripening. Taken together, our results provide new insights into the molecular mechanisms regulating biosynthesis of fatty acid and short-chain VOCs in fruit.

## Introduction

Fruit aroma, determined by a mixture of volatile organic compounds (VOCs), is an essential aspect of flavor quality that affects consumer liking. Poor flavor of modern fruit cultivars caused by the loss of important VOCs has existed for decades. There is an increasing demand among consumers for flavor improvement. An important first step to improvement is knowledge of the regulation of volatile biosynthesis pathways.

Volatiles are mainly derived from essential nutrients, including fatty acids. Polyunsaturated omega-3 (*ω*-3) linolenic acid (18:3) is the main precursor of multiple VOCs. For instance, 18:3-derived (*E*)-2-hexenal, (*Z*)-3-hexenal, and (*Z*)-3-hexenol are important high-abundance VOCs that contribute to the unique fresh note of multiple fruit species, including tomato, kiwifruit, and peach. Moreover, these C6 VOCs are also associated with defense against mechanical and herbivore damage in the plant kingdom [1, 2]. As important constituents of cellular membranes, fatty acids also serve as precursors for a wide range of metabolites. For instance, 18:3 is a substrate for synthesis of jasmonic acid, which is involved in plant immune and stress responses [3]. These observations suggest that the biosynthesis of fatty acids and their derivatives during fruit ripening is highly regulated. Fatty acid-derived short-chain VOCs are synthesized through the lipoxygenase (LOX) pathway [4], which includes LOX, hydroperoxide lyase (HPL), and alcohol dehydrogenase (ADH). These C6 alcohols can be further converted into esters through the action of alcohol acetyltransferases (AATs). Suppression of *SlLOXC*, *SlHPL*, and *SlADH2* expression resulted in reduction of fatty acid-derived VOCs in tomato fruit [5–8]. Recent studies have revealed that lipase genes are also involved in the synthesis of fatty acid-derived VOCs in tomato fruit [9, 10]. Considering the importance and abundance of 18:3-derived VOCs in multiple fruit species, it is important to understand the regulation of 18:3 synthesis and subsequent VOC production.

*De novo* fatty acid synthesis from acetyl-CoA to oleic acid (18:1) occurs in plastids. The synthesized 18:1 is further desaturated within the endoplasmic reticulum or in plastids. According to the position of the double bond inserted, fatty acid desaturases (FADs) are classified as *ω*-3 and *ω*-6 desaturases. Conversion from 18:1 to 18:2 is catalyzed by *ω*-6 FADs (FAD2 and FAD6), whereas *ω*-3 FADs (FAD3, FAD7, and FAD8) convert linoleic acid (18:2) to 18:3. FADs from many crops and fruits have been cloned and functionally characterized, including rice [11], canola [12], soybean [13], maize [14], tobacco [15], olive [16], and tomato [17, 18]. Production of seed fatty acids can be regulated by transcription factors (TFs) [19–24]. A study in *Brassica napus* revealed that conditional expression of *BnLEC1* and *BnL1L* increases 18:3 content [25]. A comprehensive assay showed that in association with LEC1-LIKE (L1L) and NUCLEAR FACTOR-YC2 (NF-YC2), the BASIC LEUCINE ZIPPER 67 (bZIP67) TF regulates biosynthesis of 18:3 by activating *FAD3* expression during seed maturation [26]. Mutation of *WRKY6* increased the expression of *FAD3* and 18:3 content in *Arabidopsis* [27]. In avocado fruit, ten expressed *AP2*/*ERF* genes related to fatty acid accumulation during ripening were identified [28]. Overall, these studies demonstrate the importance of transcriptional regulation for modification of fatty acid profiles in plants. Although TFs regulating 18:3 production have been identified in plant seeds [25–27], TFs that can affect *FAD* transcription and 18:3 biosynthesis in fruit remain undetermined.

Peach (*Prunus persica* L. Batsch) is a major world-wide commercial fruit crop. The global production of peaches reached ~25.7 million tonnes in 2019 (FAOSTAT, http://www.fao.org/faostat/en). More than 100 volatile compounds have been identified in ripe peach fruit, many of which are derived from fatty acid precursors [29]. We previously showed that peach *FAD* genes were temporally regulated during fruit development and ripening [30]. Among the four *ω*-3 *FADs* of peach fruit, expression of *PpFAD3-1* increased during fruit development and ripening [30]. We also found that peach TF PpNAC1 functionally complements the ripening deficiency of the tomato *nonripening* (*nor*) mutant [31]. Peach PpNAC1 regulates the synthesis of volatile esters by activating *PpAAT1* expression [31], which catalyzes the final step in ester formation. These results prompted us to study whether PpNAC1 could regulate *PpFAD3-1* to produce 18:3, the precursor for the downstream VOC synthesis in peach fruit. We further investigated possible epigenetic modifications associated with peach VOC production [31, 32].

Here, we show that ripe fruit-specific expression of *PpFAD3-1* is positively correlated with 18:3 content and its derived VOCs. The TF PpNAC1 activated expression of *PpFAD3-1* by directly binding to its transcriptional promoter. Correlation between gene expression and 18:3 contents across peach cultivars validated the roles of these two genes in fatty acid production. Moreover, transgenic tomato fruits were generated by overexpression of *PpFAD3-1* or *PpNAC1* to examine their roles in the synthesis of 18:3 and its derivative VOCs. We confirmed PpFAD3-1 function in producing 18:3. Metabolite and gene expression analyses were performed to evaluate the potential role of *PpNAC1* in increasing fatty acid flux towards downstream volatiles in fruit. An association between epigenetic modifications and gene expression was also investigated during peach fruit ripening. Taking these results together, we propose a model for the regulation of fatty acid-derived VOCs in fruit.

## Results

### *PpFAD3-1* expression is correlated with increased 18:3 content during peach fruit ripening

**Figure 1:**
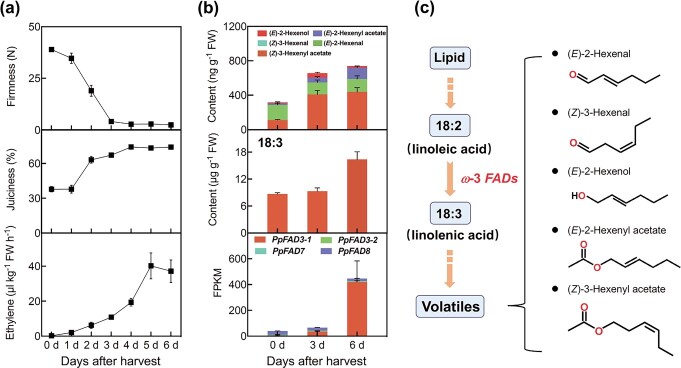
Changes in content of 18:3 and fatty acid-derived VOCs and expression of *ω*-3 *PpFADs* during peach fruit ripening. **a** Firmness, juiciness, and ethylene production. **b** Content of 18:3, its derived volatiles, and transcripts of *PpFAD*s. **c** 18:3 is a precursor for the synthesis of volatiles. Data represent the average of three independent biological replicates with standard error indicated by vertical lines.

In melting peach fruit ‘Hujingmilu’, firmness decreased rapidly from 42.7 N at harvest to 4.8 N after 3 days of postharvest ripening at 20°C (Fig. 1a), and then remained constant. Softening of the fruit was accompanied by increased juiciness after harvest. The climacteric rise of ethylene production was initiated at ~3 days after harvest, peaked at ~5 days during postharvest storage, and then declined from 6 days onward (Fig. 1a). Content of aroma VOCs derived from fatty acid increased during fruit ripening (Fig. 1b). Here, we found that increased fruit VOCs were concomitant with 18:3 content (Fig. 1b and c).

Biosynthesis of 18:3 is catalyzed by *ω*-3 FADs using 18:2 as substrate (Fig. 1c). A total of four *ω*-3 *FADs* were identified in the peach genome [30]. We compared their transcript profiles during postharvest storage at ambient temperature. We found that *PpFAD3-1* had the highest transcript levels at 6 days after harvest, accounting for ~93.9% of the *ω*-3 FAD family expression at that point in fruit maturation (Fig. 1b). *PpFAD3-1* transcripts increased nearly 251-fold during peach fruit ripening. Similarly, expression of *PpFAD3-1* increased rapidly for peel tissue during postharvest storage using real-time quantitative PCR (qPCR) analysis [30]. Therefore, we concluded that *PpFAD3-1* was the most likely candidate gene responsible for 18:3 biosynthesis during fruit ripening.

### *PpFAD3-1* contributes to the synthesis of 18:3 both *in vitro* and *in vivo*

**Figure 2:**
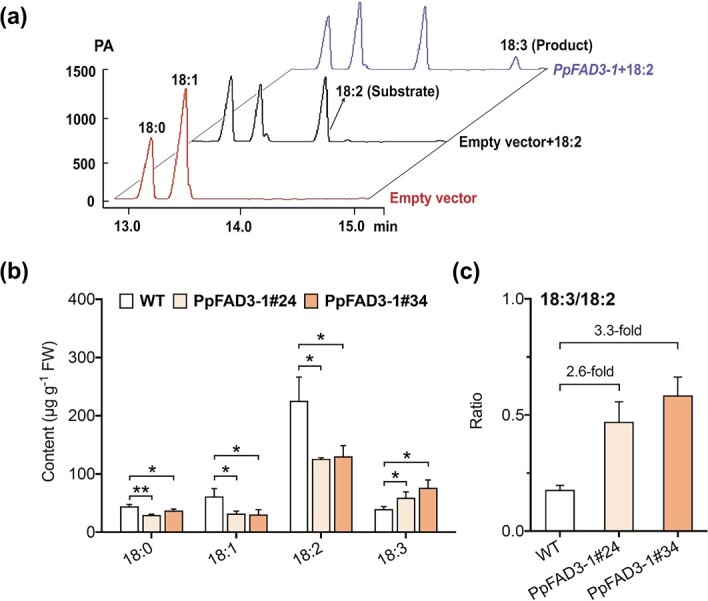
PpFAD3-1 catalyzes synthesis of 18:3 both *in vitro* and *in vivo.***a** Recombinant PpFAD3-1 converted 18:2 to 18:3 PA is the output signal of the GC instrument, which is the current ampere. **b** Fatty acids in transgenic tomato fruit overexpressing *PpFAD3-1*. **c** Ratio of 18:3/18:2. Data represent the average of three independent biological replicates with standard error indicated by vertical lines. Significant differences between transgenic fruit and WT are indicated by asterisks (^*^P < .05, ^**^P < .01).

To investigate whether PpFAD3-1 could catalyze the biosynthesis of 18:3, a full-length coding sequence of *PpFAD3-1* was cloned into the pYES2 NT/C vector and overexpressed in yeast strain INVSc1. An empty-pYES2 control did not produce 18:2 or 18:3 (Fig. 2a, bottom red line). No 18:3 was detected for empty vectors even after feeding with 18:2 as substrates (Fig. 2a, middle black line). For recombinant PpFAD3-1 protein, production of 18:3 was observed after feeding 18:2 substrates (Fig. 2a, upper blue line). These *in vitro* results demonstrated that PpFAD3-1 enzyme could convert 18:2 into 18:3.

To our knowledge, no reports of transgenic peach fruit exist. Therefore, we overexpressed *PpFAD3-1* in tomato to determine if it contributes to 18:3 production. Two independent transgenic lines (PpFAD3-1#24 and PpFAD3-1#34) were generated for analysis. Transgenic tomato fruits contained significantly higher levels of 18:3, with increases up to ~200% compared with the wild type (WT) (Fig. 2b). Overexpressing *PpFAD3-1* also resulted in significant decreases in 18:2 contents (Fig. 2b). As a consequence of the overexpression, the ratio of 18:3/18:2 significantly increased in transgenic tomato fruits (Fig. 2c). A similar increase in 18:3 and reduction in 18:2 content was also observed in transgenic tomato leaves (Supplementary Data Fig. S1). Therefore, overexpressing *PpFAD3-1* contributes to the synthesis of 18:3 *in vivo*. Taken together, our results indicated that *PpFAD3-1* is associated with 18:3 production*.*

### Effect of *PpFAD3-1* overexpression on fatty acid-derived VOCs and expression of genes for volatile synthesis

We further investigated whether overexpressing *PpFAD3-1* could alter volatile production in transgenic tomato fruit, particularly for 18:3-derived (*E*)-2-hexenal and (*Z*)-3-hexenol. Transgenic tomato fruit showed a marked decrease in the amount of 18:2-derived volatiles, including hexanal and hexanol (Table 1). Levels of hexanal in fruit from line PpFAD3-1#24 exhibited an ~53% decrease compared with the WT fruit (Table 1). Reduction of hexanal and hexanol was associated with a decrease in 18:2 content (Fig. 2b). Although 18:3 was increased significantly after overexpressing *PpFAD3-1* (Fig. 2b), no significant changes were observed for (*E*)-2-hexenal and (*Z*)-3-hexenol in fruit from either of two independent lines (Table 1). The increase in the VOC (18:3)/VOC (18:2) ratio was mainly caused by the decline of 18:2-derived volatiles after overexpressing *PpFAD3-1* rather than an increase in 18:3-derived volatiles.

**Table 1:** Contents of fatty acid derived volatiles (ng g^−1^ FW) in transgenic tomato fruit overexpressing *PpFAD3-1*.

**Volatiles**	**Precursor**	**WT**	**Transgenic tomato fruit line**	
			**PpFAD3-1#24**	**PpFAD3-1#34**
Hexanal	18:2	1034.67 ± 150.25^a^	476.16 ± 68.47^b^	484.68 ± 92.52^b^
Hexanol	18:2	55.93 ± 16.45^a^	17.92 ± 4.58 c	31.52 ± 5.83^b^
(*E*)-2-Hexenal	18:3	3024.99 ± 222.70^a^	2417.96 ± 171.28^a^	3402.58 ± 135.92^a^
(*Z*)-3-Hexenal	18:3	155.37 ± 32.14^b^	405.69 ± 75.17^a^	75.67 ± 16.25^b^
(*Z*)-3-Hexenol	18:3	139.67 ± 36.99^a^	85.32 ± 7.36^b^	162.90 ± 16.41^a^
VOC (18:3)/VOC (18:2) ratio		3.08 ± 0.26	5.73 ± 0.85	7.40 ± 1.09

Data represent the average of three independent biological replicates with standard error. ANOVA using Duncan’s test was used to calculate the differences between transgenic fruit and WT. Means with different letters are significantly different at *P* < .05.

**Figure 3:**
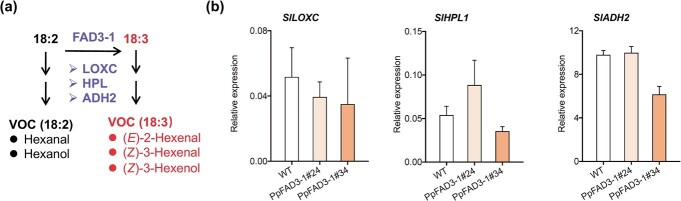
Expression of genes involved in synthesis of volatiles in transgenic tomato fruit overexpressing *PpFAD3-1*. **a** Pathway for the synthesis of fatty acid-derived volatiles. **b** Expression of *SlLOXC*, *SlHPL*, and *SlADH2* in transgenic tomato fruit. Data represent the average of three independent biological replicates with standard error indicated by vertical lines.

These results prompted us to analyze the expression of genes associated with volatile synthesis in tomato fruit. C6 volatiles derived from fatty acids are formed by the LOX pathway. In tomato fruit, *SlLOXC*, *SlHPL*, and *SlADH2* are three genes involved in LOX-mediated oxidation of unsaturated fatty acids 18:2 and 18:3, contributing to the formation of VOCs (Table 1). Compared with WT, no significant changes in transcript levels were observed for *SlLOXC*, *SlHPL*, and *SlADH2* in the transgenic tomato fruit (Fig. 3b). Our data do not permit us to assign a role for *PpFAD3-1* in the synthesis of fruit volatiles since we cannot exclude a possible difference in LOX pathway between peach and tomato fruit. However, for transgenic tomato fruit, ectopic expression of peach *PpFAD3-1* is not sufficient to enhance the production of 18:3-derived volatiles; activation of the LOX pathway must also be required. Given the roles of various TFs in enhancing secondary metabolite accumulation in plants, it would be interesting to determine whether there are specific TFs that regulate the production of both 18:3 and VOCs in fruit.

### Overexpressing *PpNAC1* increased content of 18:3 and its derived VOCs

**Figure 4:**
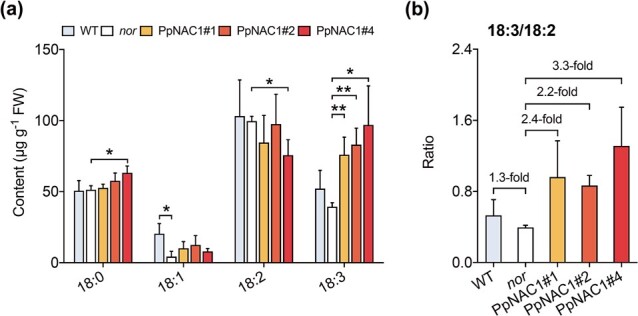
Fatty acid profiles in transgenic tomato fruit overexpressing *PpNAC1*. **a** Content of fatty acids. **b** Ratio of 18:3/18:2. Data represent the average of three independent biological replicates with standard error indicated by vertical lines. Significant differences between transgenic fruit and WT are indicated by asterisks (^*^P < .05, ^**^P < .01).

The contents of 18:3 and its derived VOCs increased during fruit ripening (Fig. 1b and c). We speculated that synthesis of the genes encoding biosynthetic enzymes responsible for these chemicals is activated by a ripening-related TF. Our previous study demonstrated that TF PpNAC1 has high homology with tomato NAC-NOR [31], a key regulator for tomato fruit ripening [33, 34]. Thus, *PpNAC1* was overexpressed in the tomato *nor* mutant to study whether *PpNAC1* could restore 18:3 production in fruit. Three independent transgenic lines were generated for analysis: PpNAC1#1, PpNAC1#2, and PpNAC1#4. As expected, transgenic tomato fruit produced a 2.5-fold increase in 18:3 levels compared with the *nor* mutant (Fig. 4a). No significant reduction in 18:2 was observed in the three independent transgenic tomato lines. Compared with WT fruit, the transgenic tomato plants overexpressing *PpNAC1* had a 3.3-fold increase in the 18:3/18:2 ratio (Fig. 4b).

**Figure 5:**
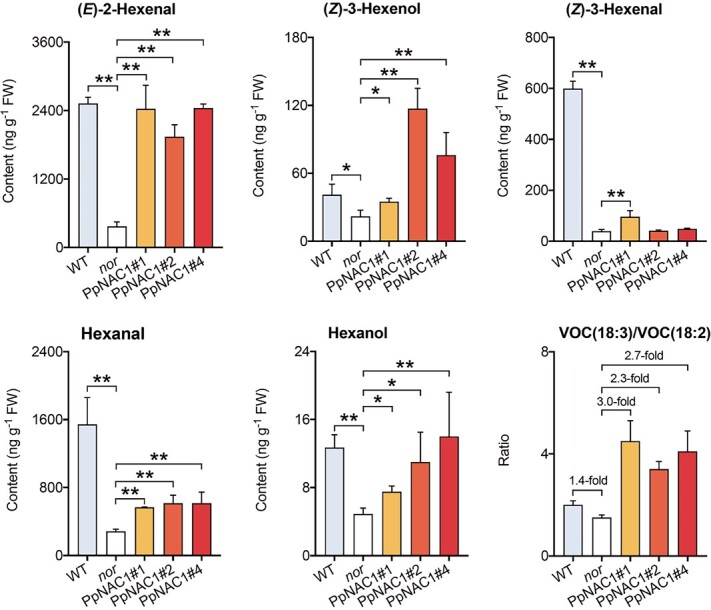
Fatty acid-derived volatiles in transgenic tomato fruit overexpressing *PpNAC1*. Data represent the average of three independent biological replicates with standard error indicated by vertical lines. Significant differences between transgenic fruit and WT are indicated by asterisks (^*^P < .05, ^**^P < .01).

The *nor* mutant tomato fruit had significantly lower contents of volatiles than the WT. Overexpressing *PpNAC1* in the *nor* mutant resulted in significantly increased contents of 18:3-derived (*E*)-2-hexenal and (*Z*)-3-hexenol, similar to the levels in WT fruit (Fig. 5). Moreover, the content of 18:2-derived hexanal and hexanol increased by 2.2- and 2.8-fold, respectively. Consequently, the VOC (18:3)/VOC (18:2) ratio increased in transgenic tomatoes, in agreement with the increased 18:3/18:2 ratio (Fig. 4b). These results indicated that overexpressing *PpNAC1* positively affected 18:3 accumulation and enhanced the biosynthesis of 18:3-derived short-chain C6 volatiles in fruit.

**Figure 6:**
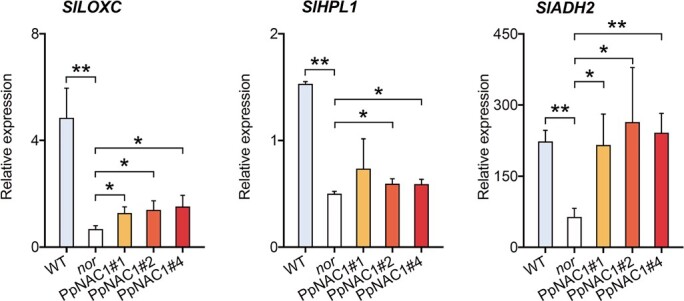
Transcript levels of genes responsible for volatile synthesis in transgenic tomato fruit overexpressing *PpNAC1*. Data represent the average of three independent biological replicates with standard error indicated by vertical lines. Significant differences between transgenic fruit and WT are indicated by asterisks (^*^P < .05, ^**^P < .01).

Real-time qPCR was applied to study changes in gene expression caused by overexpressing *PpNAC1*. Compared with WT tomato fruit, transcript levels of *SlLOXC*, *SLHPL*, and *SlADH2* were significantly reduced in the *nor* mutant. Transgenic fruit overexpressing *PpNAC1* had significantly higher *SlLOXC*, *SLHPL*, and *SlADH2* contents (Fig. 6). Thus, increased levels of transcripts of these genes, together with higher 18:3 content, contributed to the enhanced production of (*E*)-2-hexenal and (*Z*)-3-hexenol. Overexpressing *PpNAC1* in fruit also led to a significant increase in expression of *SlFAD3* (Supplementary Data Fig. S2), which is associated with the conversion of 18:2 to 18:3 [35]. Increased transcript levels of *SlLOXC*, *SlHPL*, and *SlADH2* caused by overexpressing *PpNAC1* were also accompanied by increased contents of volatiles involving 18:2-derived hexanal and hexanol (Fig. 5).

### PpNAC1 transcriptionally regulates *PpFAD3-1* by directly binding to its promoter

**Figure 7:**
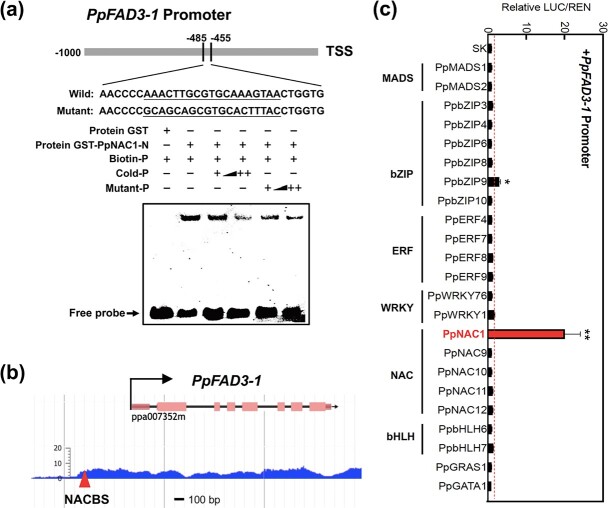
TF PpNAC1 activates *PpFAD3-1* and binds to its promoter. **a** EMSA of PpNAC1 binding to the *PpFAD3-1* promoter. **b** ChIP-Seq of *PpFAD3-1* with PpNAC1. The position of NACBS was identified by EMSA. **c** Dual-luciferase reporter assay in tobacco (*N. benthamiana*). The LUC/REN ratio of the empty vector (SK) plus *PpFAD3-1* promoter was set as 1. Error bars represent standard error (*n* = 6). ^**^P < .01.

Given that overexpressing *PpNAC1* or *PpFAD3-1* could enhance the production of 18:3, we next investigated if PpNAC1 could activate *PpFAD3-1* expression. To study whether PpNAC1 can directly bind to the promoter of *PpFAD3-1*, an electrophoretic mobility shift assay (EMSA) was performed. Recombinant GST-PpNAC1 protein was produced (Supplementary Data Fig. S4). Two probes containing two NAC binding sites (NACBSs) were designed and 3′-labeled with biotin. The EMSA results showed that only the biotin probe containing the sequence (T/A)NN(C/T)(T/C/G)TNNNNNNNA(A/C)GN (A/C/T)(A/T) bound to the promoter of *PpFAD3-1* (Fig. 7a). The binding decreased when the concentration of cold probe (as competitor) increased. When the predicted binding sites were mutated, binding was eliminated. We also determined that the position of a ChIP-Seq peak associated with the *PpFAD3-1* gene is consistent with the position of the NACBS identified from the EMSA results (Fig. 7b). These results demonstrated that PpNAC1 directly binds to the *PpFAD3-1* promoter both *in vivo* and *in vitro*.

**Figure 8:**
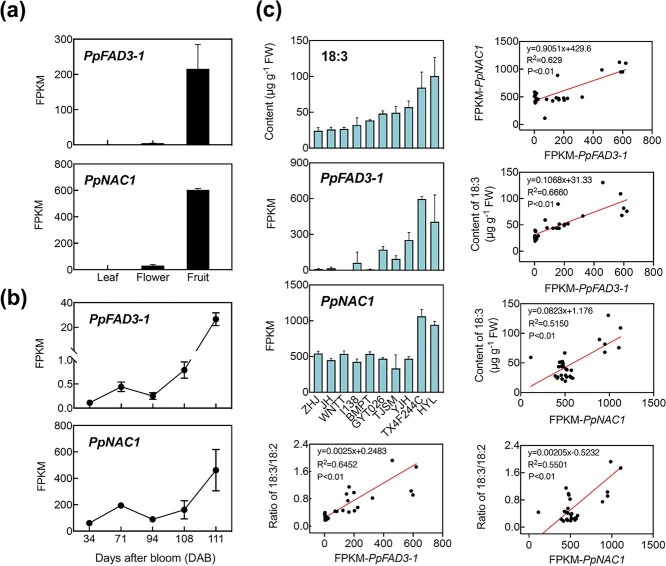
Correlation of 18:3 and transcript levels of *PpNAC1* and *PpFAD3-1* in fruit. **a** Transcript levels of *PpFAD3-1* and *PpNAC1* in leaf, flower, and fruit. **b** Transcripts of *PpFAD3-1* and *PpNAC1* during fruit development and ripening. **c** Correlations between content of 18:3, 18:3/18:2 ratio, and transcript levels of *PpFAD3-1* and *PpNAC1* across 10 peach cultivars. Error bars represent standard error (*n* = 3).

We next performed dual-luciferase reporter assays in tobacco (*Nicotiana benthamiana*). An ~20-fold induction of *PpFAD3-1* was observed with TF PpNAC1 (Fig. 7c). According to previous studies [36, 37], two NACBSs were predicted to be located in the *PpFAD3-1* promoter (Supplementary Data Fig. S3). We also investigated if other TFs could activate *PpFAD3-1* transcription. TFs with potential binding sites and positive correlations (*R* > .5, *P* < .05) in expression patterns during fruit ripening were characterized, including members of the TF bZIP, WRKY, MYB, NAC, ERF, MADS, and bHLH families (Supplementary Data Table S1). Among these TFs, PpNAC1 had the strongest induction of *PpFAD3-1* (Fig. 7c). In addition, both *PpFAD3-1* and *PpNAC1* exhibited the highest expression levels in ripe fruit compared with other organs, including leaf and flower (Fig. 8a). Also, the transcript levels of *PpNAC1* positively correlated with *PpFAD3-1* during fruit development and ripening (*R* = .94, *P* < .05) (Fig. 8b). Our observations demonstrated that PpNAC1 activates the expression of *PpFAD3-1* by directly binding to its promoter.

### Expression of *PpNAC1* correlates with 18:3 content across peach cultivars

In order to further explore the role of *PpNAC1* in regulating 18:3 contents in peach fruits, natural variation of gene expression and 18:3 content was determined across 10 peach cultivars (Fig. 8c). The results of a linear regression analysis showed that the expression of *PpNAC1* and *PpFAD3-1* and the content of 18:3 positively correlated across cultivars. Transcript levels of *PpNAC1* were significantly and positively correlated with 18:3 contents (*R*^2^ = .515, *P* < .01) (Fig. 8c) and transcripts of *PpFAD3-1* (*R*^2^ = 0.629, *P* < .01). Moreover, a positive correlation was also observed between the expression of *PpNAC1* and the ratio of 18:3/18:2 in fruit (*R*^2^ = .550, *P* < .01). Our present results indicated that PpNAC1 was a candidate TF to regulate *PpFAD3-1* expression and the synthesis of 18:3 in fruit.

### Changes in epigenetic modifications of *PpFAD3-1* and *PpNAC1* during fruit ripening

Having identified roles for *PpFAD3-1* and *PpNAC1* in the synthesis of 18:3 and its derived volatiles, we next explored whether expression of these genes is associated with epigenetic modifications during fruit ripening. Our previous study showed *PpNAC1* expression was associated with changes in histone methylation [31]. Therefore, a dataset derived from the fruitENCODE project [38] was used to analyze any epigenetic modifications for *PpFAD3-1*.

**Figure 9:**
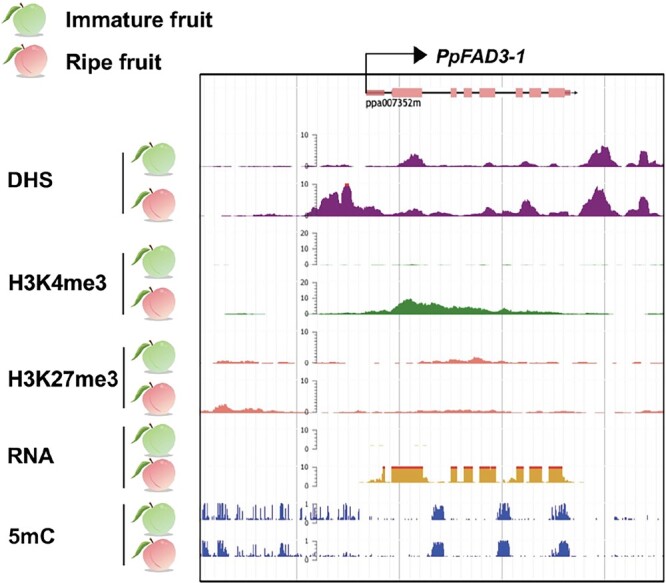
Epigenetic modifications of *PpFAD3-1* during fruit ripening. Data were accessed from the fruitENCODE project database.

DNase I hypersensitive sites (DHSs) are genomic regions containing active *cis*-regulatory DNA elements. Fruit ripening induced a large number of DHSs (peak signals) in the promoter regions of *PpFAD3-1* (Fig. 9), accompanied by high transcript levels as fruit ripen. Chromatin immunoprecipitation (ChIP)-Seq results confirmed that PpNAC1 binds to the *PpFAD3-1* promoter (Fig. 9). The location of the NACBS used in the EMSA was consistent with the position of a ChIP-Seq peak. Moreover, this NACBS was over-represented within DHS peaks. These results indicate that the *PpFAD3-1* promoter becomes more open and accessible during fruit ripening, facilitating binding of PpNAC1 to the *PpFAD3-1* promoter *in vivo*.

As fruits ripened, *PpFAD3-1* H3K4me3 marker (active epigenetic mark) was increased (Fig. 9), while the repressive histone mark H3K27me3 was removed (Fig. 9). The methylome dataset showed a widespread distribution of DNA methylation in the promoter of *PpFAD3-1*, while decreased 5mC levels were detected in the *PpFAD3-1* promoter as fruit ripened (Fig. 9).

**Figure 10:**
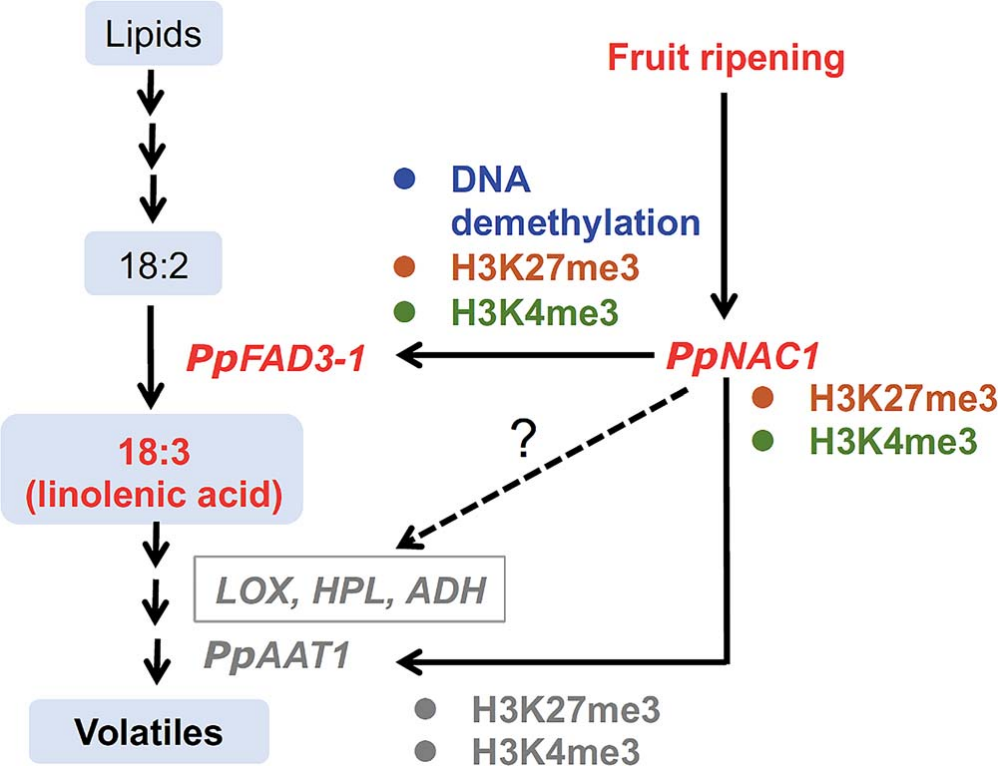
A proposed model for the synthesis of 18:3 and volatiles during fruit ripening.

These results for *PpFAD3-1*, together with our previous studies of *PpNAC1* and *PpAAT1* [31], suggest a model for the regulation of 18:3 synthesis and subsequent volatile production during peach fruit ripening (Fig. 10). PpNAC1 activates ripening-related *PpFAD3-1* expression via directly binding to its promoter, which in turn catalyzes 18:3 formation in fruit. Histone modifications, including increased active mark H3K4me3 and decreased repressive mark H3k27me3, provide an extra level of regulation for gene expression and fatty acid production as the fruit ripens. Increased expression of *PpFAD3-1* is associated with demethylation in its promoter during ripening. Therefore, activation of the metabolic pathway for the synthesis of 18:3 and volatiles is associated with transcriptional regulation and epigenetic modifications during fruit ripening.

## Discussion

Dissatisfaction with fruit flavor has been a common consumer complaint for decades. Loss of aroma volatiles is one of the major factors contributing to the deterioration of fruit flavor quality. Understanding the molecular basis of volatile regulation in fruit is essential to restore and improve the flavor quality of fruit. Our present study provided new insights into the molecular mechanisms of flavor-related volatile synthesis.

The *ω*-3 fatty acid 18:3 is required for synthesis of short-chain VOCs that have a great impact on fruit flavor and consumer preferences. Thus, FADs that catalyze 18:3 synthesis are of great interest for modifying flavor volatile content in fruit. Here, we showed that overexpressing *PpFAD3-1* in tomato fruit caused a significant increase in the ratio of 18:3-derived VOCs/18:2-derived VOCs. Similarly, transgenic tomato fruit produced an increase in the 18:3/18:2 ratio and (*Z*)-3-hexenal/hexanal after overexpression of *B. napus BnFAD3* and potato *StFAD7* [35]. Despite greater accumulation of 18:3 after overexpressing *PpFAD3-1*, it is noteworthy that we did not observe a significant increase in 18:3-derived VOCs in tomato fruit, including (*E*)-2-hexenal and (*Z*)-3-hexenol. Fatty acids are converted into C6 volatiles through the downstream LOX pathway, which includes LOX, hydroperoxide lyase, and alcohol dehydrogenase [5–7]. Overexpressing *PpFAD3-1* did not alter *SlLOXC*, *SlHPL*, or *SlADH2* transcript levels. Therefore, simultaneous activation of LOX pathway genes will also be required to increase C6 volatile content in fruit. To test this hypothesis, *PpNAC1* was overexpressed in tomato fruit, resulting in significant increases in *SlLOXC*, *SLHPL*, and *SlADH2* transcripts. Notably, production of 18:2-derived VOCs increased by ~220%, although the content of precursor 18:2 did not increase significantly. In addition to precursor fatty acids, our results underscore the importance of activating expression of volatile biosynthetic genes.

The importance of TFs for regulating the formation of metabolites has been demonstrated previously. For instance, structural genes involved in the anthocyanin synthesis pathway can be activated by MYBs in many plants [39, 40]. Here, results of dual-luciferase assays, EMSAs, and ChIP-Seq confirmed that PpNAC1 can positively regulate *PpFAD3-1* expression by directly binding to its promoter both *in vitro* and*in vivo*. A previous study demonstrated that PpNAC1 was the closest homologue of the tomato NAC-NOR [31], which is an essential regulator of fruit ripening. In a CRISPR/Cas9-edited *slnor* tomato mutant [34], fruit transcripts of *SlLOXC*, *SLHPL*, and *SlADH2* were significantly decreased. AATs responsible for the final step in fatty acid-derived ester synthesis were also regulated by NACs [31]. Our present study, together with previous results, demonstrates that *PpNAC1* functionally complements the *NAC-NOR* loss of function and restores the pathway for the biosynthesis of 18:3 and its derived volatiles. Thus, PpNAC1 has a major role in modulating fatty acid flux to produce more flavor volatiles during fruit ripening.

In addition to transcriptional regulation, epigenetic modifications are also associated with gene expression. Compared with unripe fruit, the methylation levels of activation mark H3K4me3 increased within both the *PpFAD3-1* and *PpNAC1* [31] promoter regions, while the methylation levels of repressive mark H3K27me3 tended to decrease. Histone modifications are associated with *FAD3* expression and the synthesis of 18:3 in *Arabidopsis* seeds [41] and banana fruit [42]. Moreover, increased expression of *PpFAD3-1* also accompanied decreased DNA methylation levels in the promoter regions in fruit. Apart from functioning as a precursor for synthesis of flavor volatiles in fruit, the 18:3 *ω*-3 fatty acid is also essential for biosynthesis of signaling molecules, including jasmonic acid [3]. It is known that increased 18:3 is required to maintain membrane fluidity for plants undergoing temperature stress [43]. Moreover, increased 18:3 has been suggested to contribute to lipid oxidation, which in turn contributes to tissue breakdown during fruit ripening and senescence [44]. Given the important roles of fruit 18:3, multiple levels of regulation for *PpFAD3-1* expression can be expected.

In summary, our study provides new insights into the molecular mechanisms regulating essential fatty acid 18:3 production as well as synthesis of volatiles derived from it. Metabolic engineering of VOCs in tomato fruit revealed an important role for PpNAC1 in activating *PpFAD3-1* expression to provide *ω*-3 fatty acid for subsequent steps in the volatile synthesis pathway. Moreover, transcription of *PpNAC1* and *PpFAD3-1* is associated with epigenetic modifications during fruit ripening. This valuable information will facilitate the manipulation of fatty acid composition as well as improvements in fruit flavor quality.

## Materials and methods

### Plant materials and sampling

Peach (*P. persica* L. Batsch cv. ‘Hujingmilu’) fruits were harvested from an orchard at Ningbo, Zhejiang Province. Fruits were transferred to the laboratory within 3 hours after harvest, and then stored at 25°C for 6 days for postharvest ripening. Fruits at different developmental stages were harvested and sampled as previously described [45]. Fruits of 10 cultivars, comprising ‘Zaohujing’ (ZHJ), ‘Juhuang’ (JH), ‘Weinantiantao’ (WNTT), ‘I138’ (I138), ‘Baimangpantao’ (BMPT), ‘GYT026’ (GYT026), ‘Tianjinshuimi’ (TJSM), ‘Yejihong’ (YJH), ‘TX4F244C’ (TX4F244C), and ‘Huiyulu’ (HYL) were harvested at ripe stage. Fruit mesocarp slices were combined, frozen in liquid nitrogen and stored at −80°C until use. Three biological replicates with five fruits each were used in the present study.

### Fatty acid extraction and analysis

Extraction of fatty acids was performed as previously described [30]. Firstly, 2 g of frozen tissue powder was mixed with 15 ml of *n*-hexane:isopropanol (3:2, v/v) and 7.5 ml of 6.7% Na_2_SO_4_, followed by centrifugation for 10 minutes. The supernatant was evaporated to dryness with nitrogen. Methanol:toluene:H_2_SO_4_ (88:10:2, v/v/v) was added to produce fatty acid methyl esters (FAMEs). After cooling, 1 ml of heptane with 0.5 g anhydrous Na_2_SO_4_ was added for FAME extraction. To detect fatty acids, an Agilent 6890 N gas chromatograph equipped with a flame ionization detector and a DB-WAX column (0.25 mm, 30 m, 0.25 $\upmu$m; J & W Scientific) was used. The injector and detector temperatures were 230°C. The initial oven temperature was 50°C, increased to 200°C at 25°C min^−1^, then increased to 230°C at 3°C min^−1^. Nitrogen was used as the carrier gas at 1 ml min^−1^. Exogenous heptadecanoic acid was added as internal standard.

### Volatile extraction and gas chromatography–mass spectrometry analysis

Volatiles were extracted and analyzed as previously described [46]. Frozen tissues were ground into powder, and 5 g of tissue was added into a vial containing 3 ml of 200 mM ethylenediaminetetraacetic acid (EDTA) and 3 ml of 20% CaCl_2_. The vials were placed in the tray of a solid-phase micro-extraction (SPME) autosampler (Combi PAL, CTC Analytics, Agilent Technologies, USA) coupled to an Agilent 7890A gas chromatograph and an Agilent 5975C mass spectrometer. For volatile collection, a polydimethylsiloxane and divinylbenzene (PDMS-DVB) (Supeclo Co., Bellefonte, PA) fiber was used. The extracted volatiles were separated by a DB-WAX column (30 m × 0.25 mm i.d. × 0.25 μm film thickness; J & W Scientific, Folsom, CA). Temperature started at 40°C, increased to 100°C at 3°C min^−1^, and then to 245°C at 5°C min^−1^. The column effluent was ionized by electron ionization at an energy of 70 eV. Volatiles were identified by comparing their electron ionization mass spectra with the NIST Mass Spectral Library (NIST-08) and the retention time of authentic standards. Quantification of volatiles was performed using the peak area of the internal standard as a reference.

### RNA extraction and gene expression analysis

The protocol described in our previous study [44] was used to extract total RNA. Real-time qPCR was performed with the SsoFast™ EvaGreen Supermix Kit and CFX96 instrument (Bio-Rad, Hercules, CA, USA). Primers are listed in Supplementary Data Table S2. Three independent replicates of RNA extraction and cDNA synthesis were used for qPCR analysis. RNA sequencing (RNA-Seq) was performed on an Illumina HiSeq 2500 sequence platform as previously described [31]. Three biological replicates for each ripening stage were performed. After removing adapter reads, ambiguous reads, and inferior quality reads, ~1.55 Gb clean reads were produced, with 91.92% mapped to the peach genome. Paired reads were mapped to the peach genome (https://phytozome-next.jgi.doe.gov/, *P. persica* v2.1), and data analysis was performed as described in our previous study [47]. Transcript abundance was expressed as RPKM (reads per kilobase of exon model per million mapped reads) based on the length of the gene and the number of reads mapped to this gene.

### Eukaryotic expression and enzymatic activity assay

A full-length coding sequence of *PpFAD3-1* was cloned and inserted into the pYES2 NT/C vector using the primers listed in Supplementary Data Table S2. Empty pYES2 NT/C vector was used as a control. The vectors were transformed to *Saccharomyces cerevisiae* strain INVSc1. Single yeast colonies were selected, added to 1 ml of SD-Ura + glucose culture solution and cultured at 30°C, 250 rpm until the OD_600_ reached 0.4–0.5. Mixtures were then centrifuged and dissolved for fatty acid analysis using by gas chromatography.

### Dual-luciferase assays

According to a previous protocol [31], the fragment of the *PpFAD3-1* promoter was inserted into the pGreen II 0800-LUC vector using the primers listed in Supplementary Data Table S2. For TFs, full cDNAs were inserted into the pGreen II 0029-SK vector with the primers listed in Supplementary Data Table S1. The recombinant vectors were electroporated into *Agrobacterium tumefaciens* GV3101. A mixture of 1 ml TFs and 100 μl promoter was prepared to infiltrate into tobacco leaves (*N. benthamiana*) using 1-ml needleless syringes. The tobacco leaves were mashed with 1 × PBS. Enzyme activities of firefly luciferase (LUC) and *Renilla* luciferase (REN) were measured using a Modulus luminometer (Promega, Madison, WI, USA). Three independent experiments with six biological replicates each were performed for each TF–promoter interaction.

### Electrophoretic mobility shift assay

The full-length open reading frame (ORF) of PpNAC1 was inserted into the pGEX-4 T-1 vector using the primers listed in Supplementary Data Table S2. Purified protein of PpNAC1 was used to perform EMSA, with the Lightshift™ Chemiluminescent EMSA kit (Thermo Fisher Scientific, New York, NY, USA). DNA fragments in the promoter of *PpNAC1* containing the NACBS were labeled with biotin and then annealed to yield the double-strand biotin-labeled probes. A competitor of unlabeled DNA fragment was used as the cold probe while a mutated sequence of NACBS was used as the mutation probe. The details of the EMSA experiment were described in our previous study [31].

### Stable overexpression in tomato

A full-length cDNA of *PpFAD3-1* was cloned and inserted into the pBI121 vector containing a CaMV35S promoter with primers listed in Supplementary Data Table S2. Tomato transformation was performed as previously described [31]. Transgenic lines and WT tomato plants were grown in a greenhouse. Tomato fruit at 7 days post-breaker stage (B + 7) from the T2 generation as well as WT plants were harvested. Three biological replicates with six fruits each were harvested, and then frozen in liquid nitrogen and stored at −80°C for further analysis.

### Gene epigenetic regulation analysis

The epigenetic modifications were accessed from the fruitENCODE database (https://www.ncbi.nlm.nih.gov/geo/query/acc.cgi?acc=GSE116581). The gene ID of *PpFAD3-1* for searching is ppa007352m (Prupe.6G056100).

### Statistical analysis

Figures were prepared using GraphPad Prism 8.0 (GraphPad Software, San Diego, CA, USA). Unpaired Student’s *t*-test was used to perform two-sample significance tests, while multiple comparisons were subjected to ANOVA using Duncan’s test using SPSS 26.0 (SPSS Inc., Chicago, IL, USA).

## Supplementary Material

Web_Material_uhac085

## Data Availability

The RNA-Seq raw data can be found in the NCBI with accession number PRJNA576753 for samples at different development and ripening stages. DHS, ChIP-Seq, H3K4me3, H3K27me3, and DNA methylation data were derived from the fruitENCODE project (https://www.ncbi.nlm.nih.gov/geo/query/acc.cgi?acc=GSE116581).

## References

[1] Ameye M , Allmann S, Verwaeren J et al.. Green leaf volatile production by plants: a meta-analysis. New Phytol. 2018;220:666–83. 28665020 10.1111/nph.14671

[2] McCormick AC , Unsicker SB, Gershenzon J. The specificity of herbivore-induced plant volatiles in attracting herbivore enemies. Trends Plant Sci. 2012;17:303–10. 22503606 10.1016/j.tplants.2012.03.012

[3] Howe GA , Major IT, Koo AJ. Modularity in jasmonate signaling for multistress resilience. Annu Rev Plant Biol. 2018;69:387–415. 29539269 10.1146/annurev-arplant-042817-040047

[4] Schwab W , Davidovich-Rikanati R, Lewinsohn E. Biosynthesis of plant-derived flavor compounds. Plant J. 2008;54:712–32. 18476874 10.1111/j.1365-313X.2008.03446.x

[5] Speirs J , Lee E, Holt K et al.. Genetic manipulation of alcohol dehydrogenase levels in ripening tomato fruit affects the balance of some flavor aldehydes and alcohols. Plant Physiol. 1998;117:1047–58. 9662548 10.1104/pp.117.3.1047 PMC34921

[6] Chen G , Hackett R, Walker D et al.. Identification of a specific isoform of tomato lipoxygenase (TomloxC) involved in the generation of fatty acid-derived flavor compounds. Plant Physiol. 2004;136:2641–51. 15347800 10.1104/pp.104.041608 PMC523329

[7] Shen J , Tieman D, Jones JB et al.. A 13-lipoxygenase, TomloxC, is essential for synthesis of C5 flavour volatiles in tomato. J Exp Bot. 2014;65:419–28. 24453226 10.1093/jxb/ert382 PMC3904703

[8] Goulet C , Kamiyoshihara Y, Lam NB et al.. Divergence in the enzymatic activities of a tomato and *Solanum pennellii* alcohol acyltransferase impacts fruit volatile ester composition. Mol Plant. 2015;8:153–62. 25578279 10.1016/j.molp.2014.11.007

[9] Garbowicz K , Liu Z, Alseekh S et al.. Quantitative trait loci analysis identifies a prominent gene involved in the production of fatty acid-derived flavor volatiles in tomato. Mol Plant. 2018;11:1147–65. 29960108 10.1016/j.molp.2018.06.003

[10] Li X , Tieman D, Liu Z et al.. Identification of a lipase gene with a role in tomato fruit short-chain fatty acid-derived flavor volatiles by genome-wide association. Plant J. 2020;104:631–44. 32786123 10.1111/tpj.14951

[11] Wang J , Ming F, Pittman J et al.. Characterization of a rice (*Oryza sativa* L.) gene encoding a temperature-dependent chloroplast omega-3 fatty acid desaturase. Biochem Biophys Res Commun. 2006;340:1209–16. 16406238 10.1016/j.bbrc.2005.12.126

[12] Yang Q , Fan C, Guo Z et al.. Identification of FAD2 and FAD3 genes in *Brassica napus* genome and development of allele-specific markers for high oleic and low linolenic acid contents. Theor Appl Genet. 2012;125:715–29. 22534790 10.1007/s00122-012-1863-1

[13] Pereira SL , Huang YS, Bobik EG et al.. A novel omega3-fatty acid desaturase involved in the biosynthesis of eicosapentaenoic acid. Biochem J. 2004;378:665–71. 14651475 10.1042/BJ20031319 PMC1223990

[14] Mikkilineni V , Rocheford TR. Sequence variation and genomic organization of fatty acid desaturase-2 (fad2) and fatty acid desaturase-6 (fad6) cDNAs in maize. Theor Appl Genet. 2003;106:1326–32. 12677402 10.1007/s00122-003-1190-7

[15] Bhunia RK , Chakraborty A, Kaur R et al.. Enhancement of α-linolenic acid content in transgenic tobacco seeds by targeting a plastidial ω-3 fatty acid desaturase (fad7) gene of *Sesamum indicum* to ER. Plant Cell Rep. 2016;35:213–26. 26521211 10.1007/s00299-015-1880-z

[16] Contreras C , Mariotti R, Mousavi S et al.. Characterization and validation of olive FAD and SAD gene families: expression analysis in different tissues and during fruit development. Mol Biol Rep. 2020;47:4345–55. 32468255 10.1007/s11033-020-05554-9

[17] Wang HS , Yu C, Tang XF et al.. A tomato endoplasmic reticulum (ER)-type omega-3 fatty acid desaturase (LeFAD3) functions in early seedling tolerance to salinity stress. Plant Cell Rep. 2014;33:131–42. 24129846 10.1007/s00299-013-1517-z

[18] Lee MW , Padilla CS, Gupta C et al.. The *FATTY ACID DESATURASE2* family in tomato contributes to primary metabolism and stress responses. Plant Physiol. 2020;182:1083–99. 31767693 10.1104/pp.19.00487 PMC6997702

[19] Baud S , Mendoza MS, To A et al.. WRINKLED1 specifies the regulatory action of LEAFY COTYLEDON2 towards fatty acid metabolism during seed maturation in *Arabidopsis*. Plant J. 2007;50:825–38. 17419836 10.1111/j.1365-313X.2007.03092.x

[20] Mu J , Tan H, Zheng Q et al.. LEAFY COTYLEDON1 is a key regulator of fatty acid biosynthesis in *Arabidopsis*. Plant Physiol. 2008;148:1042–54. 18689444 10.1104/pp.108.126342 PMC2556827

[21] Maeo K , Tokuda T, Ayame A et al.. An AP2-type transcription factor, WRINKLED1, of *Arabidopsis thaliana* binds to the AW-box sequence conserved among proximal upstream regions of genes involved in fatty acid synthesis. Plant J. 2009;60:476–87. 19594710 10.1111/j.1365-313X.2009.03967.x

[22] Shen B , Allen WB, Zheng P et al.. Expression of *ZmLEC1* and *ZmWRI1* increases seed oil production in maize. Plant Physiol. 2010;153:980–7. 20488892 10.1104/pp.110.157537 PMC2899924

[23] To A , Joubès J, Barthole G et al.. WRINKLED transcription factors orchestrate tissue-specific regulation of fatty acid biosynthesis in *Arabidopsis*. Plant Cell. 2012;24:5007–23. 23243127 10.1105/tpc.112.106120 PMC3556972

[24] Li D , Jin C, Duan S et al.. MYB89 transcription factor represses seed oil accumulation. Plant Physiol. 2017;173:1211–25. 27932421 10.1104/pp.16.01634 PMC5291041

[25] Tan H , Yang X, Zhang F et al.. Enhanced seed oil production in canola by conditional expression of *Brassica napus* LEAFY COTYLEDON1 and LEC1-LIKE in developing seeds. Plant Physiol. 2011;156:1577–88. 21562329 10.1104/pp.111.175000 PMC3135965

[26] Mendes A , Kelly AA, van Erp H et al.. bZIP67 regulates the omega-3 fatty acid content of *Arabidopsis* seed oil by activating fatty acid desaturase3. Plant Cell. 2013;25:3104–16. 23995083 10.1105/tpc.113.116343 PMC3784602

[27] Song G , Li X, Munir R et al.. The WRKY6 transcription factor affects seed oil accumulation and alters fatty acid compositions in *Arabidopsis thaliana*. Physiol Plant. 2020;169:612–24. 32129896 10.1111/ppl.13082

[28] Ge Y , Zang X, Yang Y et al.. In-depth analysis of potential PaAP2/ERF transcription factor related to fatty acid accumulation in avocado (*Persea americana* Mill.) and functional characterization of two PaAP2/ERF genes in transgenic tomato. Plant Physiol Biochem. 2021;158:308–20. 33234384 10.1016/j.plaphy.2020.11.016

[29] Wang Y , Yang C, Li S et al.. Volatile characteristics of 50 peaches and nectarines evaluated by HP–SPME with GC–MS. Food Chem. 2009;116:356–64.

[30] Wang JJ , Liu HR, Gao J et al.. Two ω-3 FADs are associated with peach fruit volatile formation. Int J Mol Sci. 2016;17:464–74. 27043529 10.3390/ijms17040464 PMC4848920

[31] Cao X , Wei C, Duan W et al.. Transcriptional and epigenetic analysis reveals that NAC transcription factors regulate fruit flavor ester biosynthesis. Plant J. 2021;106:785–800. 33595854 10.1111/tpj.15200

[32] Wei C , Liu H, Cao X et al.. Synthesis of flavour-related linalool is regulated by PpbHLH1 and associated with changes in DNA methylation during peach fruit ripening. Plant Biotechnol J. 2021;19:2082–96. 34036730 10.1111/pbi.13638 PMC8486240

[33] Osorio S , Alba R, Damasceno CM et al.. Systems biology of tomato fruit development: combined transcript, protein, and metabolite analysis of tomato transcription factor (*nor*, *rin*) and ethylene receptor (*Nr*) mutants reveal novel regulatory interactions. Plant Physiol. 2011;157:405–25. 21795583 10.1104/pp.111.175463 PMC3165888

[34] Gao Y , Wei W, Fan Z et al.. Re-evaluation of the *nor* mutation and the role of the NAC-NOR transcription factor in tomato fruit ripening. J Exp Bot. 2020;71:3560–74. 32338291 10.1093/jxb/eraa131 PMC7307841

[35] Domínguez T , Hernández ML, Pennycooke JC et al.. Increasing omega-3 desaturase expression in tomato results in altered aroma profile and enhanced resistance to cold stress. Plant Physiol. 2010;153:655–65. 20382895 10.1104/pp.110.154815 PMC2879794

[36] Ogo Y , Kobayashi T, Itai RN et al.. A novel NAC transcription factor, IDEF2, that recognizes the iron deficiency-responsive element 2 regulates the genes involved in iron homeostasis in plants. J Biol Chem. 2008;283:13407–17. 18308732 10.1074/jbc.M708732200

[37] Zhong R , Lee C, Ye ZH. Global analysis of direct targets of secondary wall NAC master switches in *Arabidopsis*. Mol Plant. 2010;3:1087–103. 20935069 10.1093/mp/ssq062

[38] Lü P , Yu S, Zhu N et al.. Genome encode analyses reveal the basis of convergent evolution of fleshy fruit ripening. Nat Plants. 2018;4:784–91. 30250279 10.1038/s41477-018-0249-z

[39] Allan AC , Hellens RP, Laing WA. MYB transcription factors that colour our fruit. Trends Plant Sci. 2008;13:99–102. 18280199 10.1016/j.tplants.2007.11.012

[40] Espley R , Allan AC. MYBs drive novel consumer traits in fruits and vegetables. Trends Plant Sci. 2018;23:693–705. 30033210 10.1016/j.tplants.2018.06.001

[41] Wang T , Xing J, Liu X et al.. Histone acetyltransferase general control non-repressed protein 5 (GCN5) affects the fatty acid composition of *Arabidopsis thaliana* seeds by acetylating fatty acid desaturase3 (FAD3). Plant J. 2016;88:794–808. 27500884 10.1111/tpj.13300

[42] Song C , Yang Y, Yang T et al.. MaMYB4 recruits histone deacetylase MaHDA2 and modulates the expression of ω-3 fatty acid desaturase genes during cold stress response in banana fruit. Plant Cell Physiol. 2019;11:2410–22. 10.1093/pcp/pcz142 31340013

[43] Iba K . Acclimative response to temperature stress in higher plants: approaches of gene engineering for temperature tolerance. Annu Rev Plant Biol. 2002;53:225–45. 12221974 10.1146/annurev.arplant.53.100201.160729

[44] Zhang B , Chen K, Bowen J et al.. Differential expression within the LOX gene family in ripening kiwifruit. J Exp Bot. 2006;57:3825–36. 17032731 10.1093/jxb/erl151

[45] Wu B , Cao X, Liu H et al.. UDP-glucosyltransferase PpUGT85A2 controls volatile glycosylation in peach. J Exp Bot. 2019;70:925–36. 30481327 10.1093/jxb/ery419 PMC6363097

[46] Zhang B , Shen J-Y, Wei W-W et al.. Expression of genes associated with aroma formation derived from the fatty acid pathway during peach fruit ripening. J Agric Food Chem. 2010;58:6157–65. 20415420 10.1021/jf100172e

[47] Zhang B , Tieman DM, Jiao C et al.. Chilling-induced tomato flavor loss is associated with altered volatile synthesis and transient changes in DNA methylation. Proc Natl Acad Sci USA. 2016;113:12580–5. 27791156 10.1073/pnas.1613910113 PMC5098663

