# Supplementary material for: Peach fruit PpNAC1 activates PpFAD3-1 transcription to provide ω-3 fatty acids for the synthesis of short-chain flavor volatiles
Source: Hortic Res. 2022 Apr 4;9:uhac085. doi: 10.1093/hr/uhac085 (PMC9172071; doi:10.1093/hr/uhac085)
Supplement: Web_Material_uhac085 [file web_material_uhac085.zip › Supplementary files 2022.2.24.docx]

**SUPLEMENT MATERIALS**

**Table S1. Primers used to construct the full-length pGreen-SK vectors.**

| Family | Peach ID | Correlation with PpNAC1 | Name | Primer-SK (5’) | Primer-SK (3’) |
| --- | --- | --- | --- | --- | --- |
| MADS | Prupe.1G531700 | 0.91359 | *PpMADS1* | agaactagtggatccATGATGAGGGAGAAGATCAA | cccctcgaggtcgacCTAGGGAAGCCCCAGTTTGA |
|  | Prupe.1G531600 | 0.68822 | *PpMADS2* | agaactagtggatccATGATGAGGAATAAGATCAA | cccctcgaggtcgacTCACCCCAGTTTGAGAGACA |
| bZIP | Prupe.1G369300 | 0.51804 | *PpbZIP3* | agaactagtggatccATGGAGGGCAGTGATGAAAC | cccctcgaggtcgacCTATTCGCTTGACTCGTGCT |
|  | Prupe.8G091600 | 0.66188 | *PpbZIP4* | agaactagtggatccATGGCTTCTTCTAGTGGGAC | cccctcgaggtcgacTCAGTAATGAAACATCTCTG |
|  | Prupe.8G267100 | 0.83529 | *PpbZIP6* | agaactagtggatccATGTCTTCAGTTCAGCAGCA | cccctcgaggtcgacTCAGCCGAGAAACATGTCGG |
|  | Prupe.5G211200 | 0.93151 | *PpbZIP8* | agaactagtggatccATGGAGCCAAATGAATCAAA | cccctcgaggtcgacTCATGGGGTACTGGAATCTT |
|  | Prupe.1G478400 | 0.82885 | *PpbZIP9* | agaactagtggatccATGCAAGAGCAAGCGACGAG | cccctcgaggtcgacCTATAAGGATCCTTCTGCAT |
|  | Prupe.2G182800 | 0.71776 | *PpbZIP10* | agaactagtggatccATGGGAAACGACGACGACGG | cccctcgaggtcgacTCAGCCAGCAGCAACTGCAT |
| ERF | Prupe.3G032300 | 0.5327 | *PpERF4* | agaactagtggatccATGTGTGGAGGTGCTATAAT | cccctcgaggtcgacTCAGAAACCTCCCCCAATCA |
|  | Prupe.8G264900 | 0.79901 | *PpERF7* | agaactagtggatccATGTGTGGCGGTGCTATTCT | cccctcgaggtcgacTTACGGAGCAGAAACGCGGT |
|  | Prupe.5G090000 | 0.83402 | *PpERF8* | agaactagtggatccATGGACATGTTCTCCGCTCA | cccctcgaggtcgacTCAGATAGAGAAACTCCATA |
|  | Prupe.2G289500 | 0.51212 | *PpERF9* | agaactagtggatccATGGATGGTTTTTGTCCTTA | cccctcgaggtcgacTTAAATCGAATAACTCCATA |
| NAC | Prupe.4G187100 | 0.94689 | *PpNAC1* | agaactagtggatccATGGAGAGCACCGACTCCTC | cccctcgaggtcgacCTATCCCAAATTGGACTCAG |
|  | Prupe.4G053300 | 0.81133 | *PpNAC9* | agaactagtggatccATGACATGGTGCAATGACTC | cccctcgaggtcgacCTATTTTCTCTCAAGCTTTC |
|  | Prupe.7G001400 | 0.87626 | *PpNAC10* | agaactagtggatccATGGAACAAAACATGGTGGT | cccctcgaggtcgacTCAGAAGTCCCACAAGCCTT |
|  | Prupe.2G202700 | 0.68364 | *PpNAC11* | agaactagtggatccATGGGAAGCAAGTGCTACAG | cccctcgaggtcgacTCAGGGGAAAATATTAAAGA |
|  | Prupe.2G320600 | 0.84878 | *PpNAC12* | agaactagtggatccATGTTGGTGGTGGATATGGC | cccctcgaggtcgacTCAGGCCATACGCACGGTTA |
| WRKY | Prupe.6G230600 | 0.58346 | *PpWRKY76* | agaactagtggatccATGGCTGTAGATTTTATGGG | cccctcgaggtcgacCTAAGACGATTCTAGAATGA |
|  | Prupe.5G117000 | 0.57333 | *PpWRKY1* | agaactagtggatccATGGATTCTAGTAAGAGCTG | cccctcgaggtcgacTTATGAGAAAATTCCTGTGG |
| bHLH | Prupe.1G173300 | 0.69932 | *PpbHLH6* | agaactagtggatccATGGGAAAAGACAGAACAGC | cccctcgaggtcgacTTATAGCTCAACTTTCATCT |
|  | Prupe.6G343400 | 0.72870 | *PpbHLH7* | agaactagtggatccATGGCGGGAAATCCGCCTGA | cccctcgaggtcgacCTATGAAGAGGAGTTTGATT |
| GRAS | Prupe.4G007500 | 0.5293 | *PpGRAS1* | agaactagtggatccATGCAAACATCTCAGAAACA | cccctcgaggtcgacTCACCTCCATGCAGAAGACG |
| GATA | Prupe.2G212900 | 0.79427 | *PpGATA1* | agaactagtggatccATGATCGGACCGAACTTCAT | cccctcgaggtcgacTCACATGTAATCCATGGAGA |

**Table S2. Primers used for gene cloning, EMSA and qPCR analysis.**

| Primers | Sequence (5’→3’) | Description |
| --- | --- | --- |
| pYES2-*PpFAD3-1*-FP | attggatccgccacc ATGGAGACTAGTGTGACCAG | pYES2 vector cloning of *PpFAD3-1* |
| pYES2-*PpFAD3-1*-RP | gccgaattcagaTTACGAGGATTTCATCTTCTC | pYES2 vector cloning of *PpFAD3-1* |
| eGFP-*PpFAD3-1*-FP | ctgcccaaattcgcgaccggtATGGAGACTAGTGTGACCAGAGGC | 35S-eGFP vector cloning of *PpFAD3-1* |
| eGFP-*PpFAD3-1*-RP | tcctttgctagtcataccggtCGAGGATTTCATCTTCTCATAAAATG | 35S-eGFP vector cloning of *PpFAD3-1* |
| pGEX-*PpNAC1*-FP | gttccgcgtggatccccggaattcATGGAGAGCACCGACTCCTC | pGEX-4T-1 vector cloning of *PpNAC1* |
| pGEX-*PpNAC1*-RP | tcagtcacgatgcggccgctcgagctaGTTGCTGGTCTTGCTATCAGCCAGC | pGEX-4T-1 vector cloning of *PpNAC1* |
| Luc-Pro*PpFAD3-1*-FP | ggtatcgataagcttAAGTTATTCGCTCGCTTGTT | pGreen-LUC vector cloning of *PpFAD3-1* promoter |
| Luc-Pro*PpFAD3-1*-RP | tggcgtcttccatggAAGGGTACCTTGAATCCACA | pGreen-LUC vector cloning of *PpFAD3-1* promoter |
| pBI121-PpFAD3-1-FP | acgggggactctagaATGGAGACTAGTGTGACCAG | pBI121 vector cloning of PpFAD3-1 |
| pBI121-PpFAD3-1-RP | accacccggggatccTTACGAGGATTTCATCTTCT | pBI121 vector cloning of PpFAD3-1 |
| Probe1-FP-biotin | AACCCCAAACTTGCGTGCAAAGTAACTGGTG | Probe for EMSA |
| Probe1-RP-biotin | CACCAGTTACTTTGCACGCAAGTTTGGGGTT | Probe for EMSA |
| Probe2-FP-biotin | AGCTTTCTGGAGCAAGTAAATGGTTTTCAA | Probe for EMSA |
| Probe2-RP-biotin | TTGAAAACCATTTACTTGCTCCAGAAAGCT | Probe for EMSA |
| Probe1-FP | AACCCCAAACTTGCGTGCAAAGTAACTGGTG | Probe for EMSA |
| Probe1-RP | CACCAGTTACTTTGCACGCAAGTTTGGGGTT | Probe for EMSA |
| Mutant-Probe1-FP | AACCCCGCAGCAGCGTGCACTTTACCTGGTG | Probe for EMSA |
| Mutant-Probe1-RP | CACCAGGTAAAGTGCACGCTGCTGCGGGGTT | Probe for EMSA |
| qPCR-*PpFAD3-1*-FP | CTAGCAGCACCAAAACGCTG | RT-qPCR of *PpFAD3-1* |
| qPCR-*PpFAD3-1*-RP | TAACTCCATTCCTCGCCACG | RT-qPCR of *PpFAD3-1* |
| qPCR-*PpTEF2*-FP | GGTGTGACGATGAAGAGTGATG | RT-qPCR of *PpTEF2* |
| qPCR-*PpTEF2*-RP | TGAAGGAGAGGGAAGGTGAAAG | RT-qPCR of *PpTEF2* |
| qPCR-*SlFAD3*-FP | AGCTTGGTAGCATAAGGGTAGC | RT-qPCR of *SlFAD3* |
| qPCR-*SlFAD3*-RP | AAACTCCCATGACCACAATCA | RT-qPCR of *SlFAD3* |
| qPCR-*SlFAD2*-FP | TGCCGGACATTGGGGTTAC | RT-qPCR of *SlFAD2* |
| qPCR-*SlFAD2*-RP | CAGATCCAGTAAAGCGGCCA | RT-qPCR of *SlFAD2* |
| qPCR-*SlFAD6-*FP | GCTCGAAACAGAAAGGCTGC | RT-qPCR of *SlFAD6* |
| qPCR-*SlFAD6*-RP | CATGAAGAGCCCCAGTGTGT | RT-qPCR of *SlFAD6* |
| qPCR-*SlFAD7*-FP | ACAATTGGCTTGTTTGGCCTC | RT-qPCR of *SlFAD7* |
| qPCR-*SlFAD7*-RP | CCGGACTTCTACCCCACAGA | RT-qPCR of *SlFAD7* |
| qPCR-*SlLOXC*-FP | GTGCAAATACCATTAAGGCTGTG | RT-qPCR of *SlLOXC* |
| qPCR-*SlLOXC*-RP | AGTCCAGTCTTATGATCAAGCTC | RT-qPCR of *SlLOXC* |
| qPCR-*SlADH2*-FP | ATGTGTCCATGATGGCTGGG | RT-qPCR of *SlADH2* |
| qPCR-*SlADH2*-RP | GGTGATGATGCAACGAAGGC | RT-qPCR of *SlADH2* |
| qPCR-*SlHPL1*-FP | AGTGAGAGACAAAGTCGGCG | RT-qPCR of *SlHPL1* |
| qPCR-*SlHPL1*-RP | ACCACAAAGAAGCTCCCCTT | RT-qPCR of *SlHPL1* |
| qPCR-*SlActin*-FP | GAAATAGCATAAGATGGCAGACG | RT-qPCR of *SlActin* |
| qPCR-*SlActin*-RP | ATACCCACCATCACACCAGTAT | RT-qPCR of *SlActin* |

**
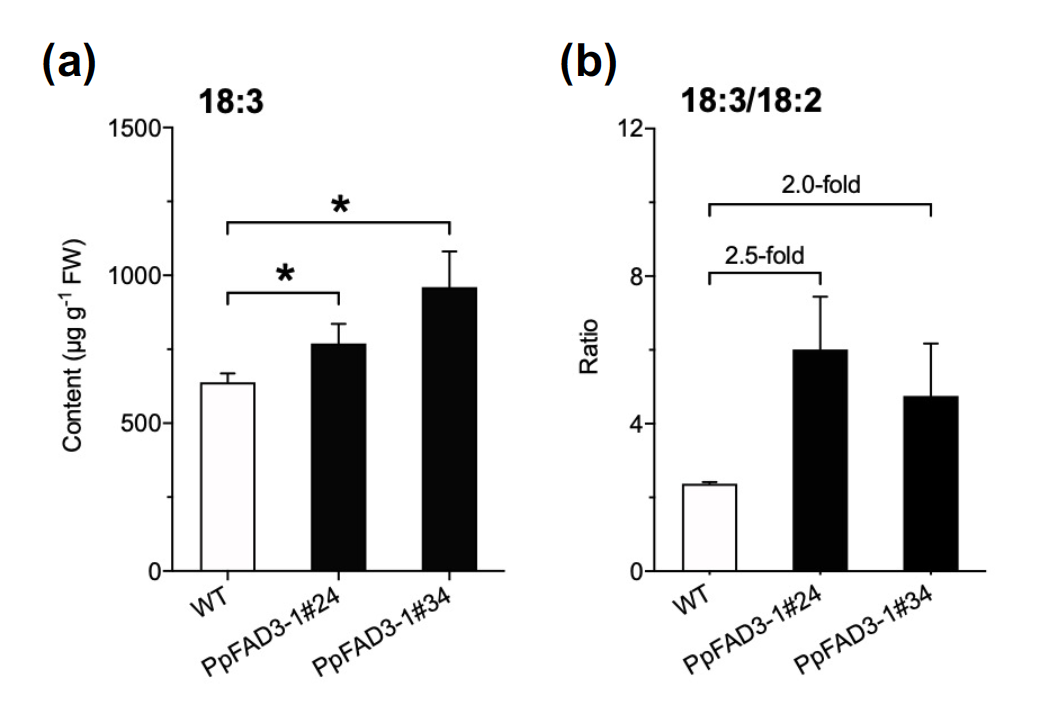
**

**Fig. S1 Contents of 18:3 and ratio of 18:3/18:2 in transgenic *PpFAD3-1* tomato leaves.** **a** Content of 18:3 in transgenic tomato leaves. **b** Ratio of 18:3/18:2 in transgenic tomato leaves. Data represent the average of three independent biological replicates with SE indicated by vertical lines. Significant differences between transgenic fruit and WT were presented with asterisks (*, *P*<0.05).

**Fig. S2 Expression of *SlFAD3* in transgenic tomato fruit.** Data represent the average of three independent biological replicates with SE indicated by vertical lines. Significant differences between transgenic fruit and WT were presented with asterisks (*, *P*<0.05, **, *P* < 0.01).

**
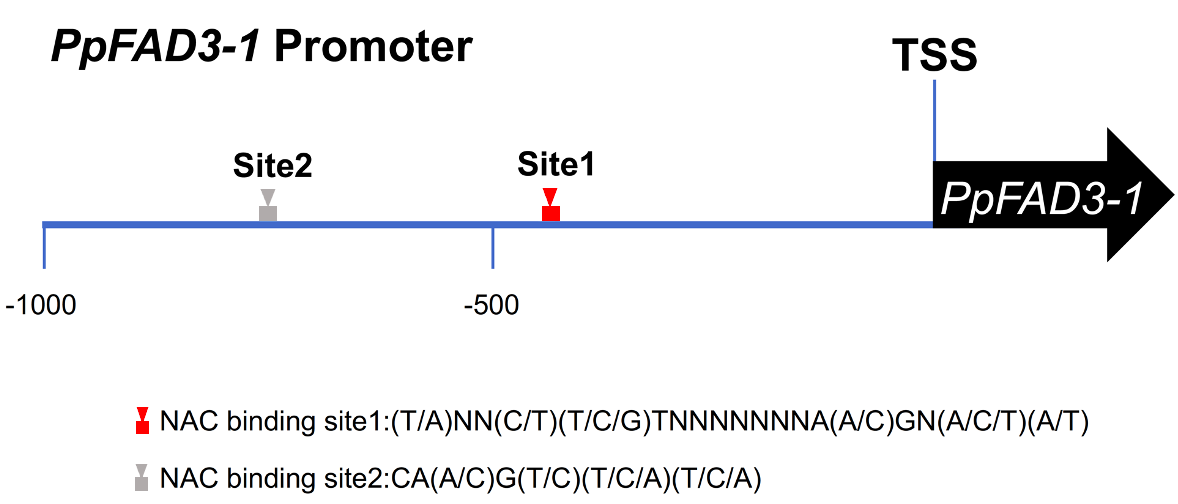
**

**Fig. S3 Two NAC protein binding sites (T/A)NN(C/T)(T/C/G)TNNNNNNNA and (A/C)GN(A/C/T)(A/T) in PpFAD3-1 promoter.**


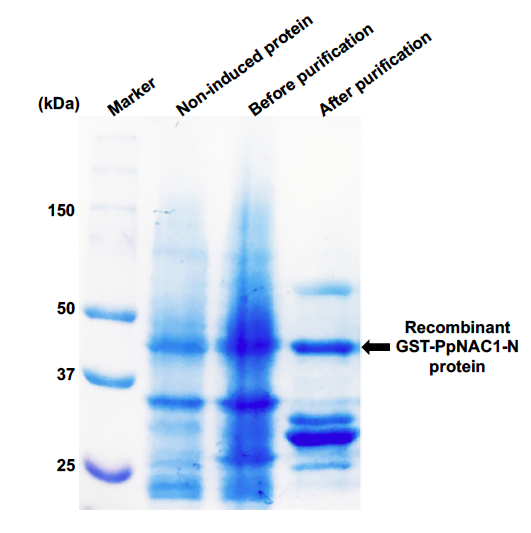


**Fig. S4 SDS-PAGE analysis of PpNAC1 protein.**
