# Supplementary figures and images for: Peach fruit PpNAC1 activates PpFAD3-1 transcription to provide ω-3 fatty acids for the synthesis of short-chain flavor volatiles
Source: Hortic Res. 2022 Apr 4;9:uhac085. doi: 10.1093/hr/uhac085 (PMC9172071; doi:10.1093/hr/uhac085)

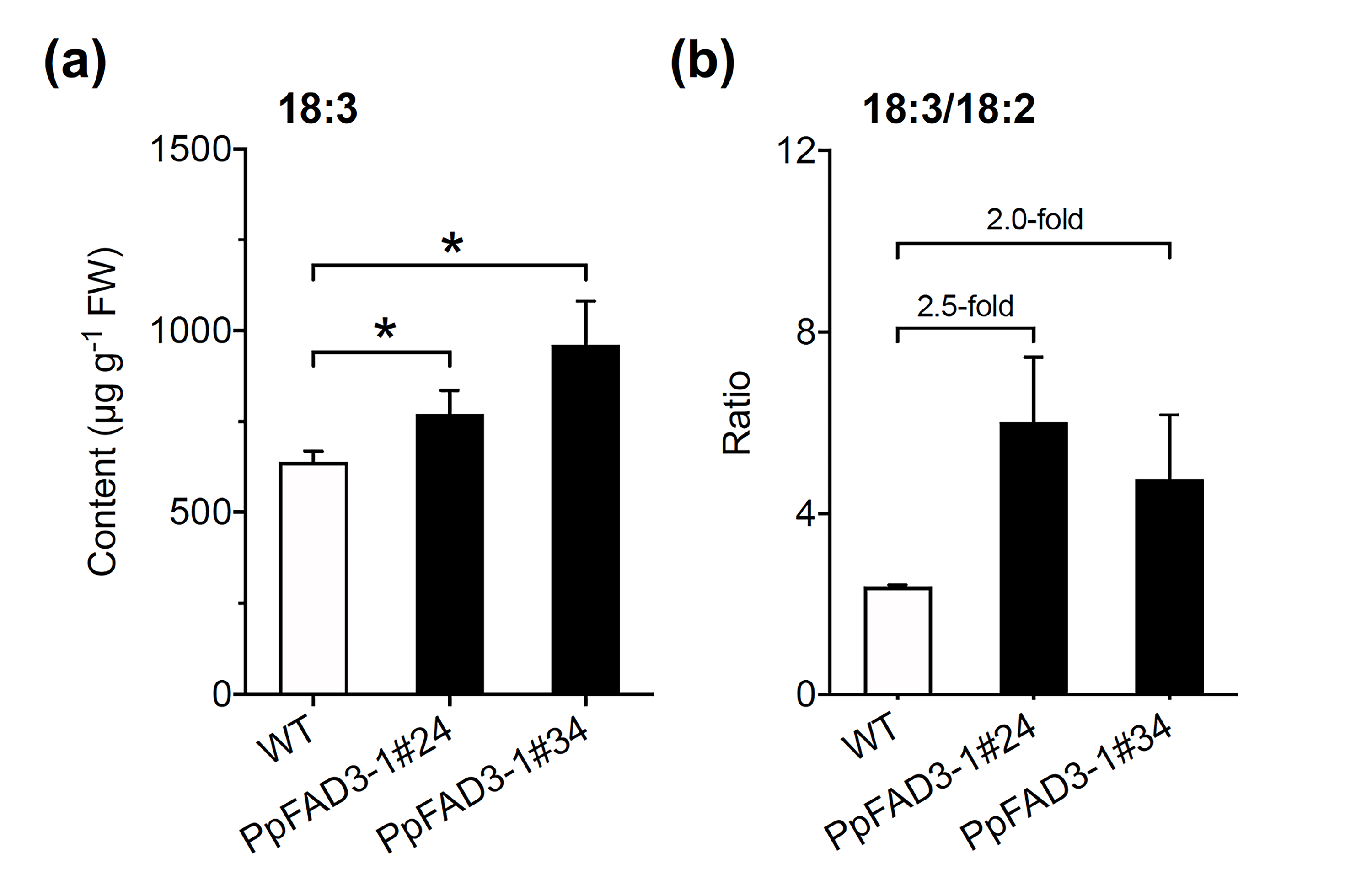

Supplement: Web_Material_uhac085 [file web_material_uhac085.zip › Figure S1.tif]

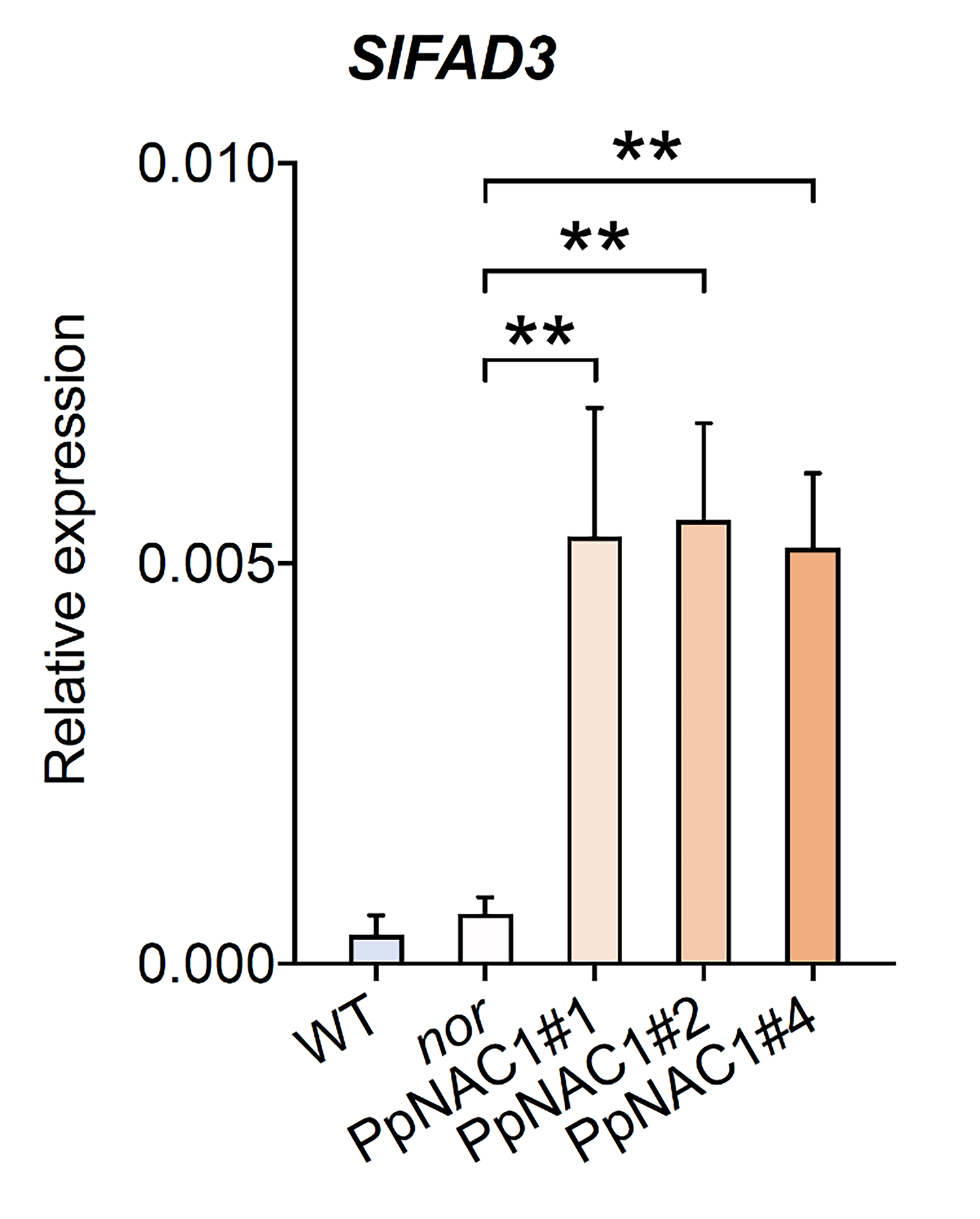

Supplement: Web_Material_uhac085 [file web_material_uhac085.zip › Figure S2.tif]

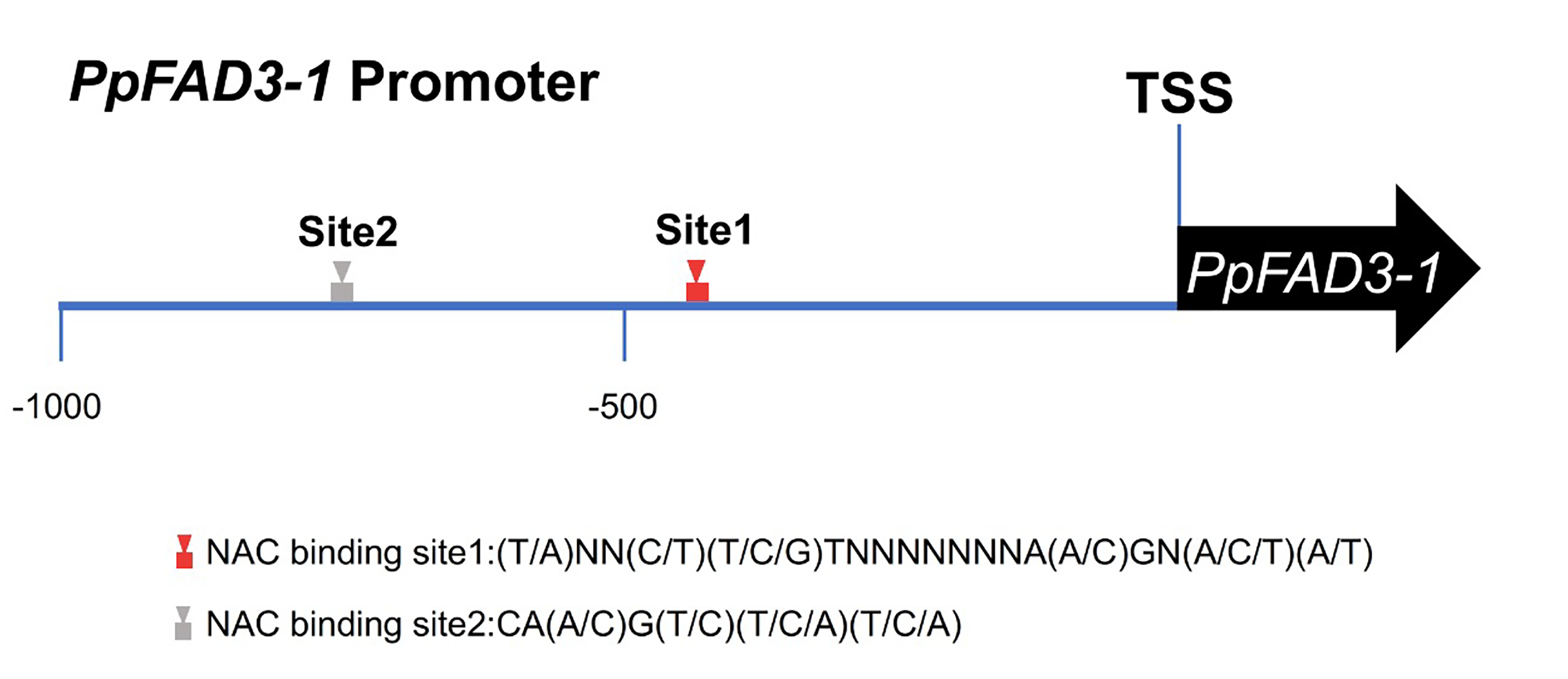

Supplement: Web_Material_uhac085 [file web_material_uhac085.zip › Figure S3.tif]

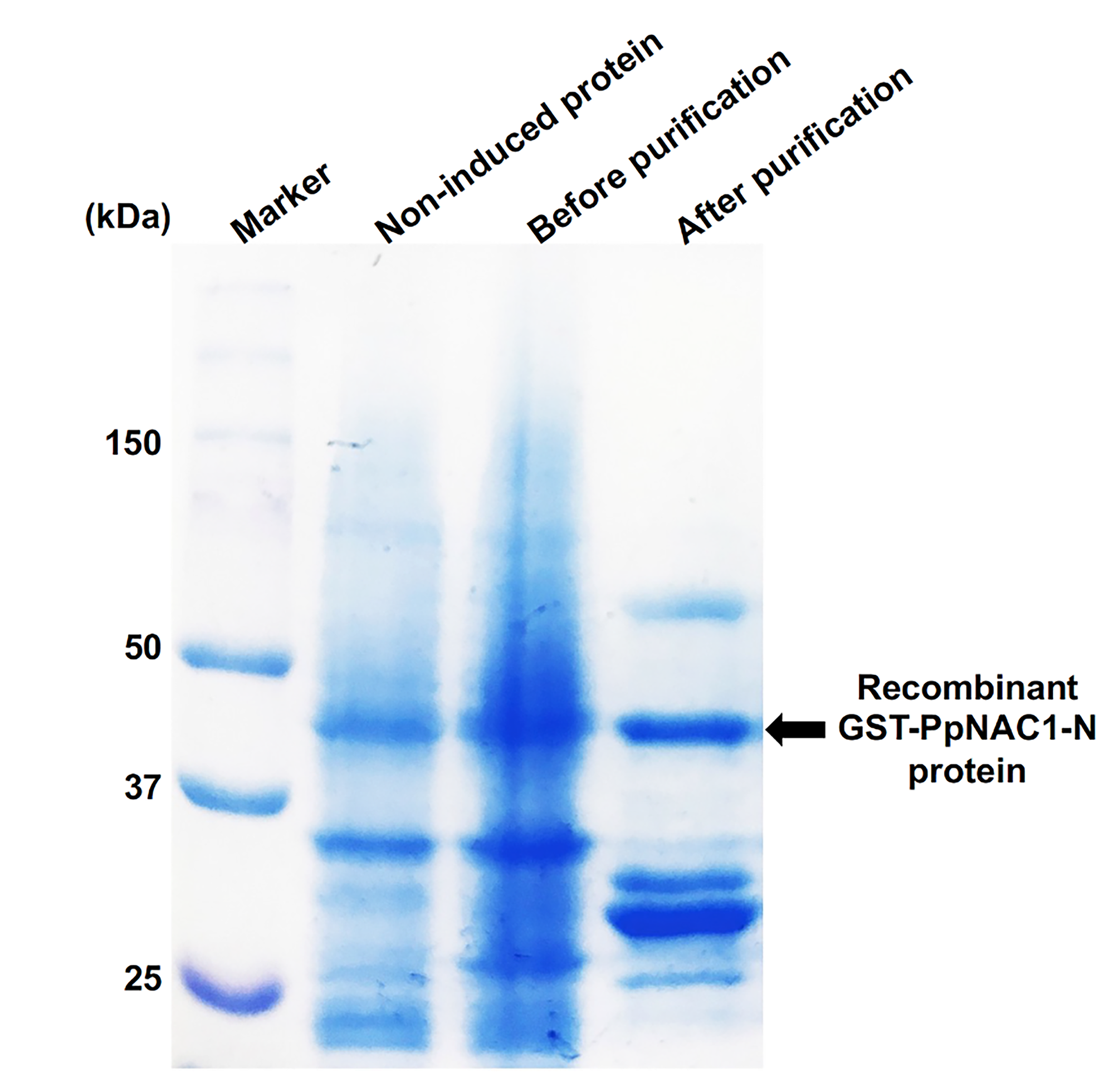

Supplement: Web_Material_uhac085 [file web_material_uhac085.zip › Figure S4.tif]
